# 832. Epidemiology, Clinical Characteristics, Treatment and Outcome of Mucormycosis during COVID-19 Pandemic in the VA Health System

**DOI:** 10.1093/ofid/ofad500.877

**Published:** 2023-11-27

**Authors:** Nirmal Muthukumarasamy, Hiroyuki Suzuki

**Affiliations:** University of Iowa Hospital and clinic, Iowa City, Iowa; University of Iowa Carver College of Medicine, Iowa City, Iowa

## Abstract

**Background:**

Mucormycosis is associated with high mortality rates, ranging between 40 to 90%. COVID-19 associated mucormycosis (CAM) has garnered much attention during the COVID-19 pandemic. As most of the reports of CAM are case reports, it is difficult to estimate the true incidence and characteristics of CAM compared to non-CAM cases. Our study aims to describe and compare CAM and non-CAM cases during the COVID-19 pandemic within the Veterans Health Administration (VHA) system.

**Methods:**

A retrospective chart review was conducted of all inpatients with a diagnostic code of mucormycosis (ICD-10: B46) among VHA hospitals from 1/1/2020 to 12/31/2022. We classified CAM as mucormycosis with a preceding COVID-19 diagnosis within 3 months. Data collected include demographics, comorbidities, COVID-19 diagnosis within 3 months, type of mucormycosis, surgical treatment and 90-day mortality.

**Results:**

During the study period, 61 patients were identified, and after a manual chart review a total of 47 patients from 29 hospitals were included in the descriptive analysis. The median age of patients was 68 years, with 46/47 (97.9%) being male. 33/46 (70.2%) were White. Most common comorbidities were diabetes mellitus (72.3%), chronic obstructive pulmonary disease (57.5%), and chronic kidney disease (40.4%). Pulmonary mucormycosis was the most common manifestation in 20 (42.6%), followed by rhino-cerebral in 15 (31.9%), cutaneous in 7 (14.9%) and musculoskeletal in 4 (8.5%) of patients.

There were 11 (23.4%) patients with CAM. Most of these cases occurred in the second half of 2021 during the Delta variant wave (8 patients), followed by 2 cases in 2022 (during the Omicron variant wave) and 1 case in 2020. More than half of patients with CAM had pulmonary disease compared with about one-third in non-CAM cases (63.6% versus 36.1%, p=0.16). CAM cases were significantly less likely to be confirmed with a biopsy (45.5% versus 80.6%, p=0.02). Mortality was higher in CAM cases (54.6% versus 30.6%, p=0.17) although the difference was not statistically significant.

Comparison of CAM and Non-CAM cases during the study period

**Figure:**
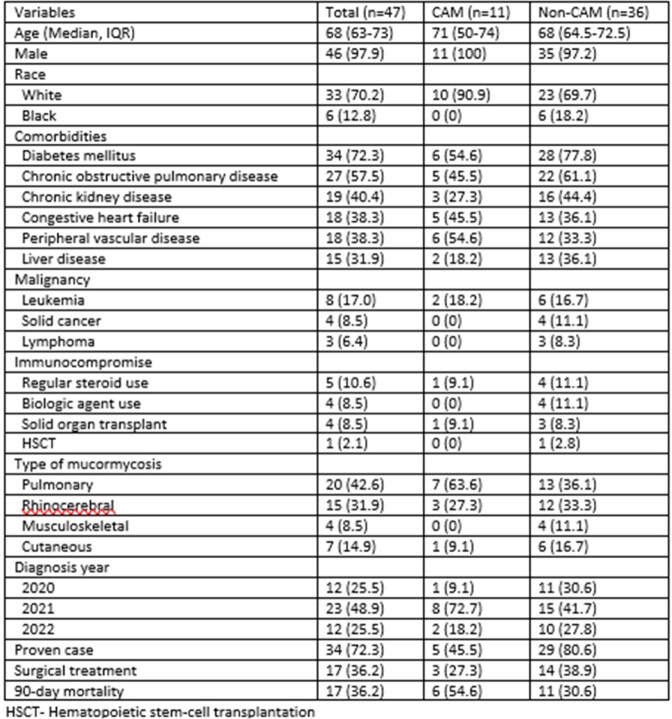

**Conclusion:**

CAM cases seemed to be quite different from non-CAM cases in clinical characteristics, treatment and outcome in our VHA cohort although a definitive conclusion cannot be made due to the limited number of cases.

**Disclosures:**

**All Authors**: No reported disclosures

